# Comparative biomarker analysis of PALOMA-2/3 trials for palbociclib

**DOI:** 10.1038/s41698-022-00297-1

**Published:** 2022-08-16

**Authors:** Zhou Zhu, Nicholas C. Turner, Sherene Loi, Fabrice André, Miguel Martin, Véronique Diéras, Karen A. Gelmon, Nadia Harbeck, Cathy Zhang, Joan Q. Cao, Zhengming Yan, Dongrui R. Lu, Ping Wei, Todd L. VanArsdale, Paul A. Rejto, Xin Huang, Hope S. Rugo, Sibylle Loibl, Massimo Cristofanilli, Richard S. Finn, Yuan Liu

**Affiliations:** 1 Pfizer Inc, La Jolla, CA USA; 2 Royal Marsden Hospital and Institute of Cancer Research, London, UK; 3 Peter MacCallum Cancer Centre, Melbourne, VIC Australia; 4 Institut Gustave Roussy, INSERM and Université Paris Saclay, Villejuif, France; 5 Instituto para la Investigación Sanitaria Gregorio Marañón, Ciberonc, Universidad Complutense, Madrid, Spain; 6 Centre Eugène Marquis, Rennes Cedex, France; 7 British Columbia Cancer, Vancouver, BC Canada; 8 Brustzentrum der Universität München (LMU), München, Germany; 9 University of California San Francisco Helen Diller Comprehensive Center, San Francisco, CA USA; 10 German Breast Group, Neu-Isenburg, Germany; 11 Weill Cornell Medicine, New York, NY USA; 12 David Geffen School of Medicine at UCLA, Santa Monica, CA USA

**Keywords:** Breast cancer, Predictive markers

## Abstract

While cyclin-dependent kinase 4/6 (CDK4/6) inhibitors, including palbociclib, combined with endocrine therapy (ET), are becoming the standard-of-care for hormone receptor–positive/human epidermal growth factor receptor 2‒negative metastatic breast cancer, further mechanistic insights are needed to maximize benefit from the treatment regimen. Herein, we conducted a systematic comparative analysis of gene expression/progression-free survival relationship from two phase 3 trials (PALOMA-2 [first-line] and PALOMA-3 [≥second-line]). In the ET-only arm, there was no inter-therapy line correlation. However, adding palbociclib resulted in concordant biomarkers independent of initial ET responsiveness, with shared sensitivity genes enriched in estrogen response and resistance genes over-represented by mTORC1 signaling and G2/M checkpoint. Biomarker patterns from the combination arm resembled patterns observed in ET in advanced treatment-naive patients, especially patients likely to be endocrine-responsive. Our findings suggest palbociclib may recondition endocrine-resistant tumors to ET, and may guide optimal therapeutic sequencing by partnering CDK4/6 inhibitors with different ETs. Pfizer (NCT01740427; NCT01942135).

## Introduction

Breast cancer is the most prevalent type of cancer in women worldwide and the second most common type of cancer overall^1^. Approximately 70% of patients have tumors positive for the estrogen receptor (ER)^2^, where endocrine therapy (ET) has been the cornerstone treatment modality. There are three main types of ET: selective ER modulators, such as tamoxifen, that inhibit the binding of ER to its ligand estrogen^3^, selective ER degraders, such as fulvestrant, that degrade or downregulate ER^4^; and aromatase inhibitors, such as letrozole, that suppress the production of estrogen^5^. Despite its clinical utility, endocrine resistance eventually emerges with all forms of ET, with patients with luminal B breast cancers at a higher risk of early relapse with ET than those with luminal A^6,7^. Although a variety of mechanisms have been implicated in the development of resistance^8,9^, most refractory patients still express ER, and its activity continues to play an important role in driving tumor growth^10^. Therefore, effective ER modulation remains essential, even in ET-resistant breast cancer.

In recent years, cyclin-dependent kinase 4/6 (CDK4/6) inhibitors have shown clinical efficacy in ER + disease when used in combination with ET. Palbociclib was the first oral selective inhibitor of CDK4/6 approved based on the PALOMA series of clinical trials^11^. PALOMA-2 was a phase 3 study of palbociclib and letrozole as first-line therapy for postmenopausal women with ER + , human epidermal growth factor receptor 2–negative (HER2–) advanced breast cancer who had no prior treatment for advanced disease^12,13^. A total of 666 women were randomly assigned in a 2:1 ratio to receive either palbociclib or placebo in combination with continuous daily letrozole. Median progression-free survival (PFS) was 27.6 months (95% CI, 22.4–30.3) in the palbociclib plus letrozole arm and 14.5 months (95% CI, 12.3–17.1) in the placebo plus letrozole arm (hazard ratio, 0.563; 95% CI, 0.461–0.687; *P* < 0.0001). Collection of OS data is still ongoing. PALOMA-3 was a phase 3 study of palbociclib plus fulvestrant in previously treated patients with advanced HR + /HER2– breast cancer who are generally younger and higher risk^14–16^. Patients were eligible if their cancer had relapsed or progressed with prior ET and the vast majority of patients (~80%) were postmenopausal. A total of 521 patients were randomly assigned in a 2:1 ratio to receive palbociclib or matching placebo in addition to fulvestrant. Median PFS was 11.2 months (95% CI, 9.5–12.9) in the palbociclib plus fulvestrant arm and 4.6 months (95% CI, 3.5–5.6) in the placebo plus fulvestrant arm (hazard ratio, 0.497; 95% CI, 0.398–0.620; *P* < 0.0001). The median OS from the updated OS analysis was 34.8 months (95% CI, 28.8–39.9) in the palbociclib plus fulvestrant group and 28.0 months (95% CI, 23.5–33.8) in the placebo plus fulvestrant group (stratified hazard ratio, 0.81; 95% CI, 0.65–0.99)^17^.

We have reported biomarker analyses from the PALOMA-2 and PALOMA-3 trials, respectively. Extensive characterization of baseline tumor tissues in PALOMA-2 using immunohistochemistry, fluorescence in situ hybridization, and gene expression panels have highlighted the importance of CDK4/6 signaling in hormone receptor–positive (HR+ )/HER2– breast cancer, revealing that the interplay between steroid hormone and peptide growth factor signaling could drive dependence on CDK4/6 signaling^18^. An analysis of baseline tumors from PALOMA-3 has identified cyclin E1 expression as a potential predictor of relative resistance to palbociclib^19^. However, a cross-therapy line comparison of biomarker patterns between the PALOMA-2 (1st line) and PALOMA-3 (2nd line or greater) is yet to be performed.

To gain further insights into the clinical benefit from adding CDK4/6 inhibitors to ET, which is becoming the standard of care in HR+ advanced or metastatic breast cancer, here we compare the gene expression/PFS relationship from the two trials in a systematic manner. Our findings from the biomarker patterns of drug response suggest the addition of palbociclib may “recondition” endocrine-resistant tumors to ET through concerted actions on CDK4/6 and ER signaling networks, with potentially important implications for achieving maximal benefit from the treatment regime and guiding the rational design of next-generation optimal therapeutic sequencing strategies.

## Results

### Discordance in predictive biomarker pattern between trials

**Fig. 1:**
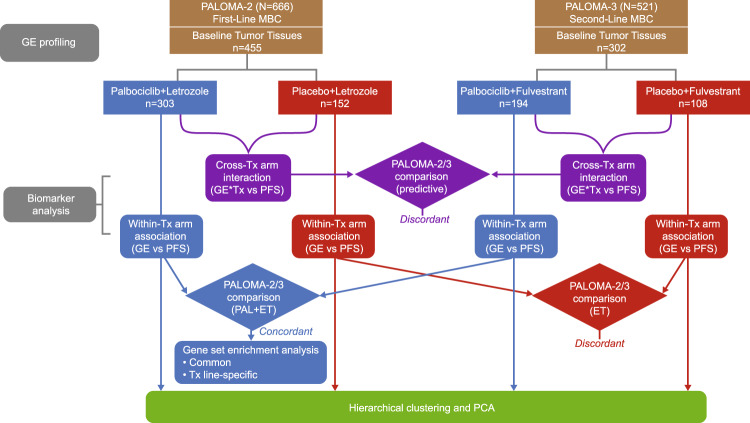
Overview of comparative PALOMA-2/3 biomarker analysis described in this study. ET endocrine therapy; GE gene expression; PAL palbociclib; PCA principal component analysis; PFS progression-free survival; MBC metastatic breast cancer; Tx treatment.

**Fig. 2:**
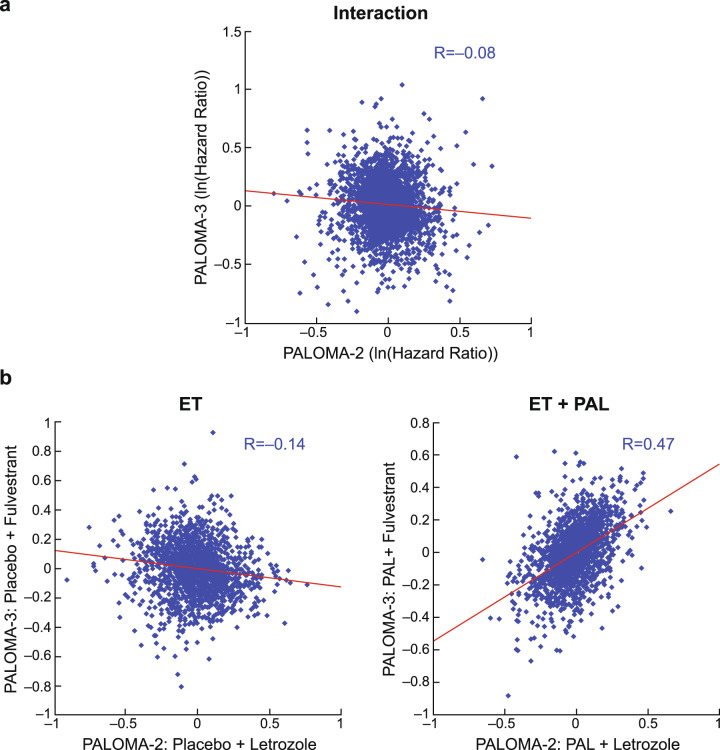
Lack of correlation in predictive biomarker pattern between PALOMA-2 and PALOMA-3 appears driven by poor concordance in the ET arm. Each data point corresponds to a gene on the EdgeSeq Oncology panel. R-value in the plots refers to Pearson correlation. **a** Comparison of gene expression/treatment effect interaction (dependency) in predicting PFS across the two trials. Plotted are interaction coefficient values (i.e. ln(Hazard Ratio)). **b** Comparison of gene expression/PFS association within each treatment arm (ET, left panel; ET plus palbociclib, right panel) across the two trials. ET endocrine therapy; PAL palbociclib; PFS progression-free survival.

A total of 455 and 302 tumors from PALOMA-2 and PALOMA-3 trials, respectively, were successfully collected and analyzed for gene expression using the EdgeSeq Oncology HTG Panel, which profiles 2534 cancer and related genes^18,19^. We first compared the predictive biomarker relationship of palbociclib benefit by examining therapy effect-by-gene expression interaction (dependency) statistic from cross-treatment arm analysis (purple, Fig. 1). The global pattern of interaction coefficient (i.e., natural log of hazard ratio) values was not conserved at all between the two trials (Pearson R = −0.08; Fig. 2a), even after restricting PALOMA-3 patients to postmenopausal ones only (Pearson R = −0.07). Among the top candidates with a nominal *P* value ≤ 0.05 (Supplementary Table 1), only 9 genes were shared between the two cohorts (enrichment *P* = 0.49), including 5 in the same direction and 4 in the opposite direction, which are essentially statistical noise.

Although discrepancy was not unexpected given the key differences in trial design such as the number of prior lines of therapy (first line vs second line or greater) and ET partner used (letrozole vs fulvestrant), we further compared the expression/PFS association pattern between PALOMA-2 and PALOMA-3 by examining the two treatment arms in each trial separately through within-arm analysis (blue and red; Fig. 1). Whereas a significant similarity was found in the combination arm (Pearson R = 0.47; *P* < 0.0001), no concordance was observed in the endocrine arm (R = −0.14; Fig. 2b), therefore the lack of correlation in the predictive biomarker pattern between the two trials observed above was driven by the endocrine arm. A similar global correlation pattern, namely good agreement in the ET + palbociclib arm and no concordance in the ET arm, was observed for hallmark gene sets representing well-defined biologic states and processes (Pearson R = 0.74 and −0.13 for combination and ET arms, respectively; Supplementary Fig. 1).

**Fig. 3:**
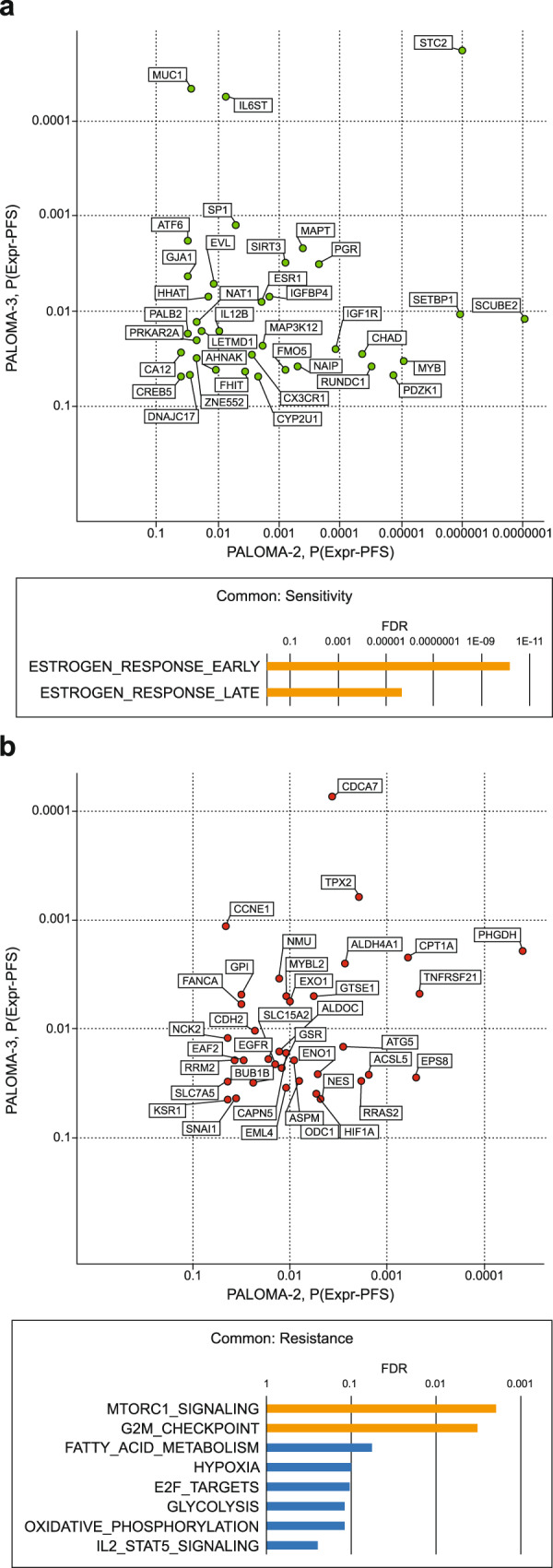
Relative consistency within the combination arm indicates a likely conserved mechanism in palbociclib response regardless of prior lines of treatment. Shown are genes whose expression are associated with PFS from treatment with palbociclib plus ET in both PALOMA-2 and PALOMA-3 (nominal *P* ≤ 0.05, top panels) and their enriched hallmark pathways (FDR ≤ 0.25, bottom panels) where orange bars highlight those with FDR ≤ 0.05. **a** Sensitivity genes whose higher expression is associated with longer PFS. **b** Resistance genes whose higher expression is associated with shorter PFS. ET endocrine therapy; FDR false discovery rate; PFS progression-free survival.

A total of 73 common genes have their expression level associated with PFS (nominal *P* ≤ 0.05) from the treatment of ET + palbociclib combination in both PALOMA-2 and PALOMA-3. Among these, 36 (e.g., *ESR1* and *PGR*) are shared sensitivity genes (i.e., have a higher expression that is associated with longer PFS; Fig. 3a), 36 (e.g., *CCNE1* and *EGFR*) are shared resistance genes (Fig. 3b), and one (*SAFB*) is inconsistent in the direction of association. The agreement far exceeds expectation by chance (*P* < 0.0001), suggesting conserved mechanisms independent of prior lines of treatment or different ET partners likely determine palbociclib response. Pathway enrichment analysis revealed that the shared sensitivity genes are dominated by those involved in estrogen response (FDR = 6.52e-11; Fig. 3a), whereas the resistance genes are overrepresented by mTORC1 signaling (FDR = 1.90e-3) and G2/M checkpoint (FDR = 3.24e-3; Fig. 3b).

### Discordance in the ET arm likely reflects key difference in ET response

In striking contrast to the ET + palbociclib arm, there is no concordance with respect to gene expression/PFS relationship in the ET arm across the two trials (Fig. 2b and enrichment *P* = 0.57 for top candidates with a nominal *P* value ≤ 0.05 shared between the two trials). This can’t be explained by differences in either endocrine therapy regimen as the letrozole arm of PALOMA-2 shares good similarity with fulvestrant+palbociclib arm of PALOMA-3 (Supplementary Fig. 2) or “hormonal milieu” as no correlation was seen even with matching menopausal status (R = −0.12, post-menopausal only). Therefore, it more likely reflects distinct ET history as second- or later-line PALOMA-3 patients had relapsed or progressed with prior endocrine treatment, whereas first-line PALOMA-2 patients had not received treatment for advanced disease. Our previous analysis of data from PALOMA-2 found that higher CDK4 expression is significantly associated with endocrine resistance (interaction *P* and ET arm association *P* = 0.0164 and *P* = 0.000972, respectively, from continuous analysis)^18^. However, no significant association was seen in the PALOMA-3 cohort (interaction *P* and ET arm association *P* = 0.16 and 0.12, respectively, from continuous analysis), where all patients were already endocrine resistant at the start of the trial unlike their advanced treatment-naive PALOMA-2 counterparts.

**Fig. 4:**
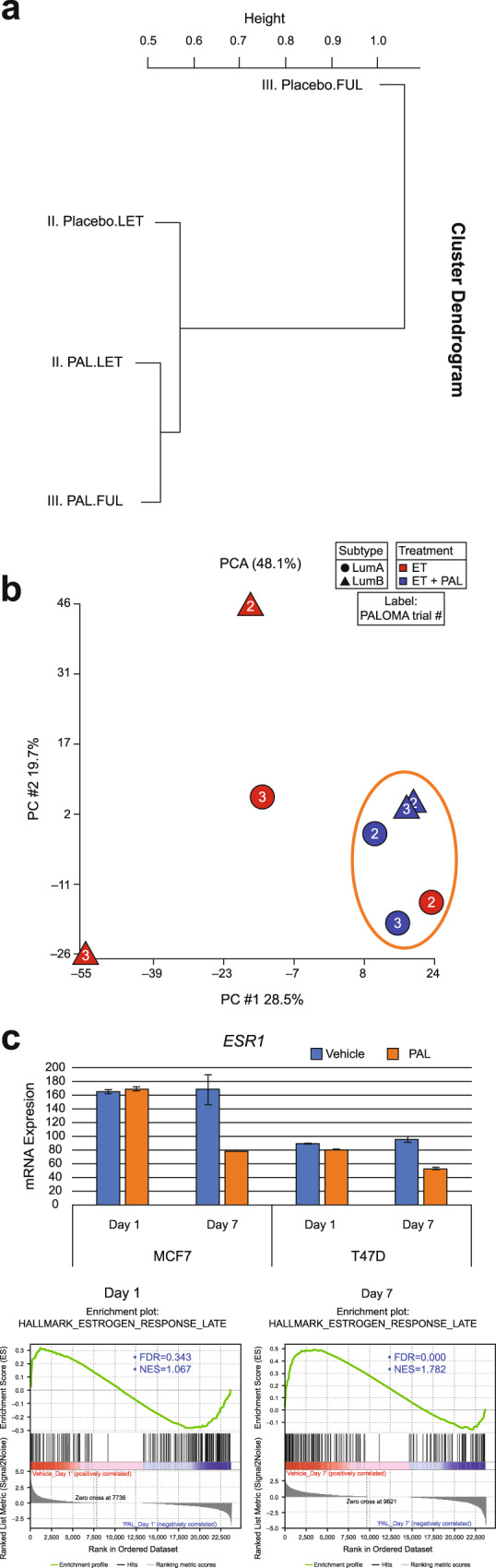
The addition of palbociclib may recondition endocrine responsiveness through concerted actions on CDK4 and ER signaling networks. **a** Hierarchical clustering analysis of gene expression/PFS association across the four treatment arms from PALOMA-2 (II) and PALOMA-3 (III). Distance metric was defined using 1 minus correlation coefficient; average linkage was used for clustering. **b** PCA of global gene expression/PFS association by molecular subtype. LumA or LumB subtype classification is represented by shape, treatment arm represented by color, and cohort represented by number in label (“2” for PALOMA-2 and “3” for PALOMA-3). The percentage values on the axes refer to the proportion of total variance accounted for by the first two principal components respectively. **c** The expression (top panel) and activity (bottom panel) of ER after palbociclib treatment in ER + cells. Transcript-level change in ESR1 on vehicle control or palbociclib at Day 1 or Day 7. GSEA enrichment plots for ER regulon (estrogen response genes) from MCF7 at Day 1 or Day 7. Genes were rank ordered from most downregulated by palbociclib (vs vehicle control) on the left to most upregulated on the right; each vertical line corresponds to a signature gene. ER estrogen receptor; ET endocrine therapy; FDR false discovery rate; FUL fulvestrant; GSEA gene set enrichment analysis; LET letrozole; LumA luminal A; LumB luminal B; NES normalized enrichment score; PAL palbociclib; PC principal component; PCA principal component analysis; PFS progression-free survival.

When we clustered Cox regression coefficient values across all four treatment arms (palbociclib plus letrozole and placebo plus letrozole from PALOMA-2; palbociclib plus fulvestrant and placebo plus fulvestrant from PALOMA-3) together, the placebo plus fulvestrant group clearly stood out as the outlier, whereas the other three were similar to each other (Fig. 4a and Supplementary Fig. 2). Given the clinical difference in ET response status between the two cohorts, the clustering of the combination arms from both trials with the treatment-naive population of PALOMA-2 suggests that the addition of palbociclib may have reconditioned endocrine-resistant patients to be more endocrine responsive. This is consistent with the pattern of ER, where its expression level, as a continuous variable, is significantly associated with clinical outcome in both treatment arms of PALOMA-2 but only in the palbociclib plus ET arm of PALOMA-3 (Supplementary Table 2).

Next we evaluated the relationship between gene expression and PFS by endocrine response status using molecular subtypes (luminal A and B) as previously described^18,19^. Notably, PCA analysis of their association pattern from Cox regression coefficient values revealed that combination treatment with palbociclib (i.e. those in blue), regardless of therapy-line setting or molecular subtype, converges toward ET of patients who are most likely to be endocrine responsive (luminal A from PALOMA-2, i.e. red circle with number 2) and away from endocrine-resistant ones (PALOMA-3, i.e. red shapes with number 3 and luminal B from PALOMA-2, i.e. red triangle with number 2; Fig. 4b). This provides further evidence that the addition of palbociclib may recondition ET responsiveness.

### Palbociclib modulates ER as a single agent in cell-line models

To gain understanding of how palbociclib may revert endocrine resistance, transcriptome changes were examined in two ER + cell lines (MCF7 and T47D) following drug treatment as previously described^20^. Cells were harvested at Day 1 and Day 7, respectively, to evaluate short- and long-term responses^20^. Interestingly, palbociclib significantly modulated ER signaling even as a single agent. Unbiased RNA-seq analysis showed that palbociclib treatment reduced not only the expression of *ESR1* but also its activity as inferred from the decreased level in estrogen-responsive genes (Fig. 4c). This effect was not evident at Day 1 but became apparent at Day 7, suggesting potential feedback from CDK to ER pathways following prolonged CDK4/6 inhibition. Therefore, palbociclib may recondition endocrine-resistant patients to ET through concerted inhibitory actions on CDK4 and ER signaling networks, both of which contribute to the development of ET resistance.

### A potentially stronger role of the T-cell–inflamed tumor microenvironment in mediating palbociclib resistance in the front-line setting

**Fig. 5:**
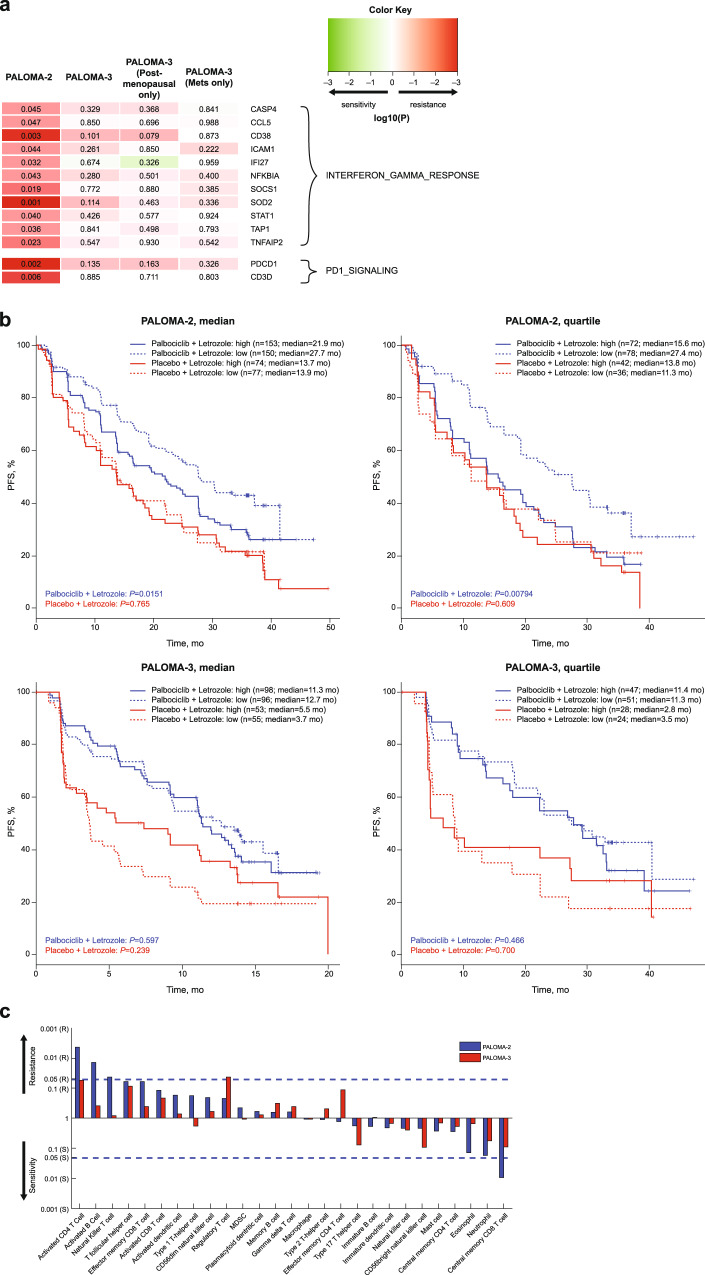
PALOMA-2–specific associations point to a potentially stronger role of the T-cell–inflamed TME in mediating palbociclib resistance in the front-line setting. **a** Gene expression/PFS association result (numeric value and color intensity represent statistical significance of the association; red = resistance, green = sensitivity) of enriched interferon-gamma response and PD1 signaling genes from palbociclib plus ET treatment in PALOMA-2, PALOMA-3, and the postmenopausal patient/metastatic sample subset of PALOMA-3, respectively. **b** Relationship of PFS and the expression of a T-cell–inflamed TME signature that has been shown to be a pan-tumor predictive biomarker for immune checkpoint inhibitor response in the clinic^23^ in PALOMA-2 (top panel) and PALOMA-3 (bottom panel) cohorts from median (left panel) and quartile (right panel) analyses. **c** Association between PFS and the expression of various immune cell types from palbociclib plus ET treatment. Shown is statistical significance in the direction of resistance (top) or sensitivity (bottom); blue lines mark *P-*value of 0.05. ET endocrine therapy; MDSC myeloid-derived suppressor cell; Mets metastatic; PFS progression-free survival; TME tumor microenvironment.

With general concordance in the gene expression-PFS relationship from palbociclib combination treatment between the two trials, there are associations restricted to specific treatment line. Interestingly, resistance genes unique to first-line (PALOMA-2) appear most enriched with interferon-gamma response (FDR = 9.30e-5; Fig. 5a), along with PD1 (*PDCD1*) and its pathway that we previously identified as a relative palbociclib resistance biomarker^18^. No such relationship was observed in PALOMA-3, even when using only samples from postmenopausal patients or metastatic samples that were collected temporally closer to the time of trial entry and thus more aligned with treatment under study (Fig. 5a). Resistance genes unique to second-line or greater (PALOMA-3) seem less coherent in terms of biological function and are most enriched with Myc targets (FDR = 0.095).

Our observation of PALOMA-2-specific PFS associations with both positive and negative immune regulatory signals may suggest a potential role of dysfunctional infiltrating phenotype typical of a T-cell–inflamed tumor microenvironment (TME)^21,22^ in mediating palbociclib resistance. We thus followed up by investigating an 18-gene signature of T-cell–inflamed TME that has been shown to be a pan-tumor predictive biomarker for immune checkpoint inhibitor response in the clinic^23,24^, 13 of which were profiled in the EdgeSeq Oncology HTG Panel (correlation of signature scores between 18-gene full set and 13-gene subset evaluated using TCGA breast cohort is 0.982, *P* < 0.0001; Supplementary Fig. 3). Higher expression of the immunotherapy biomarker signature is associated with shorter PFS from palbociclib combination treatment in PALOMA-2 (continuous association *P* = 0.000393) but not in PALOMA-3 (continuous association *P* = 0.325; Fig. 5b). It is worth noting that the relationship observed in PALOMA-2 cannot be accounted for by proliferation rate as the association remains even when slow-proliferative luminal A tumors and fast-proliferative luminal B tumors are analyzed separately (continuous association, *P* = 0.029 and *P* = 0.012, respectively). There is also no significant dependency (statistical interaction) between molecular subtype and T-cell–inflamed expression signature with respect to clinical outcome.

We further examined various immune cell types by inferring from gene expression profiles based on metagenes (i.e., nonoverlapping sets of genes that represent specific immune cell subpopulations) as previously described^25^. A total of 27 of the 28 immune cell subpopulations are represented by EdgeSeq Oncology HTG Panel genes. Consistent with the observations above, almost all significant PFS associations come exclusively from first-line PALOMA-2 patients with the only exception of regulatory T cells (Fig. 5c).

## Discussion

We present a comparative study of the gene expression and treatment outcome relationship between the PALOMA-2 and PALOMA-3 trials that reveals important insights into the potential molecular mechanism underlying clinical benefit of palbociclib. In contrast to the ET-only arm where no inter-therapy line correlation was observed at all, the addition of palbociclib appears to result in concordant biomarkers independent of initial endocrine response status, with shared sensitivity genes enriched in estrogen response and resistance genes over-represented by mTORC1 signaling and G2/M checkpoint. Furthermore, PFS/GE association patterns from combination treatment in both trials resemble that from ET in advanced treatment-naive (1st line) patients, especially among those likely to be endocrine-responsive, while distinct from refractory patients. The expression level of ER itself is also significantly associated with clinical outcome in both treatment arms of PALOMA-2 but only in the ET + palbociclib arm of PALOMA-3. Together with the observation that palbociclib can modulate estrogen receptors even on its own in ER + breast cells, our study provides, for the first time to our knowledge, phase 3 clinical trial–based evidence that palbociclib may recondition endocrine-resistant tumors to ET. This is consistent with what has been previously shown in preclinical models where palbociclib enhanced sensitivity to tamoxifen in a cell line with acquired resistance to the ET^26^ as well as our recent finding from PALOMA-2 that high CDK4 expression is linked to ET resistance, which can be mostly relieved by the addition of palbociclib^18^. It is worth noting this pattern is not apparent from the PALOMA-3 cohort where all patients were already endocrine refractory at the start of the trial.

The success of CDK4/6 inhibitors in ER + breast cancer is driven by the interplay between estrogen and CDK4/6 signaling^27^. The strong association of estrogen receptor and response with sensitivity in the palbociclib combination arms of both PALOMA-2 and PALOMA-3 supports the critical role of ER signaling in mediating clinical efficacy. With ET as the cornerstone of disease management, the development of endocrine resistance appears inevitable in the clinic, where both ER and CDK4/6 pathways are integrally involved in its escape mechanism^10,28^. CDK4/6 inhibitors such as palbociclib thus can rescue the loss of responsiveness and recondition patients to ET through concerted dual pathway inhibition.

Breast cancer tumors, especially those of ER + status, have long been considered immunologically quiescent compared with other “hot” tumor types such as melanoma and non–small cell lung cancer. Recent evidence challenges this historical notion^29,30^, and there is a growing interest in extending immunotherapy to ER + breast cancer^31–33^. The tumor-immune landscape is dynamic and heterogeneous, with significant variation observed across patients, tumor subtypes, and disease settings. Interestingly, we found a stronger role for the TME in the first-line PALOMA-2 cohort as observed from a specific association between the expression of immune genes including PD1 (*PDCD1*), and clinical outcome (PFS) from palbociclib combination treatment. The relationship was observed with both “brakes” and “accelerators” of cancer-immune response, consistent with a dysfunctional T-cell–inflamed TME^21,22^, where the infiltration of CD8 + cells and interferon-gamma secretion can upregulate local negative immunoregulatory mechanisms that reduce T-cell effector function^34,35^. We further identified a PALOMA-2–specific PFS relationship for a T-cell–inflamed signature, which has been previously shown to be a predictive biomarker of checkpoint blockade–based immunotherapy.

Immune checkpoint blockade, the most investigated form of immunotherapy in breast cancer, has been reported to achieve higher objective response rates when administered in earlier lines of therapy in multiple studies of metastatic triple-negative breast cancer (TNBC)^31,36–38^. In fact, atezolizumab in combination with nab-paclitaxel chemotherapy has been approved as front-line treatment for patients with advanced TNBC. The I-SPY2 phase 2 trial further reported that the addition of pembrolizumab to neoadjuvant chemotherapy more than doubled the estimated pathologic complete response rates for both HR + /*ERBB2*–, and triple-negative breast cancer^39^ and phase 3 studies are already ongoing. Treatments like chemotherapy or ET do not just destroy malignant cells but can significantly remodel and even induce damage to their associated TME^40,41^, which possibly makes tumors more autonomous and immune components less therapeutically relevant. The interesting observation from syngeneic mouse studies that the major factors of the CDK4/6-induced antitumor immune response are T cells^42^ provides a potential mechanistic understanding of how dysfunctional or exhausted T-cells could hinder the therapeutic effectiveness of palbociclib in a TME-dependent disease setting, which may benefit from T cell reinvigoration by immune checkpoint blockade.

While our study employs the same gene expression profiling platform and biomarker analysis protocol with both trials to enable a systematic comparison by line of therapy, it was inevitably still confounded by additional inherent cohort heterogeneity; most notably, PALOMA-2 enrolled post-menopausal patients, exclusively, while PALOMA-3 included both post- and pre/perimenopausal patients (82.5% and 17.5%, respectively, for those with biomarker data). Furthermore, tissue samples from either trial are a mixture of primary and metastatic biopsy specimen, with the source of tissue not known for PALOMA-2. We attempted to address this limitation by confirming the observed differences using a post-menopausal or metastatic subset of PALOMA-3 cohort whenever appropriate/feasible. The observed concordances, on the other hand, are less prone to heterogeneity and in fact, likely even more robust with them. Additionally, gene expression data were never directly compared between the two trials in our study but only used for association with corresponding clinical outcome within the design of each trial for exploring biomarker relationship of treatment effect (Fig. 1), whereas all inter-trial comparisons are subsequently based on these treatment-dependent association measures.

The clinical capability of palbociclib in restoring ET-resistant tumors to an endocrine responsive–like state may inform a potential optimal treatment sequencing by partnering palbociclib with different ETs to maximize the duration of therapeutic response and minimize the emergence of endocrine resistance. It also raises the exciting possibility that patients who had already progressed on previous line(s) of ET may still benefit from the same treatment by incorporating palbociclib. Further studies such as the ongoing TREnd trial^43^ are needed to directly evaluate these rational designs of therapeutic strategies, which are unlikely to be applicable exclusively to palbociclib but pertinent to other CDK4/6 inhibitors as well.

## Materials and methods

### Samples

Patients from the PALOMA-2 and PALOMA-3 clinical studies provided written consent to submit formalin-fixed paraffin-embedded (FFPE) tumor samples to assess biomarkers associated with sensitivity and/or resistance to palbociclib plus letrozole/fulvestrant per protocol. Whenever possible, recently biopsied tissue samples from a metastatic or recurrent tumor lesion were submitted. In cases where archival tissue was not available, a new biopsy sample was required. The source of tumor biopsies was documented in PALOMA-3^19^, but not in PALOMA-2^18^. Tumor content was assessed based on the percentage of malignant cells versus normal cells, and necrosis was assessed based on the percentage of total tissue area that was necrotic versus nonnecrotic. Macrodissection was performed on the tissue sections if tumor content was <70% or necrosis was ≥20%.

### Ethics

At each study center for the PALOMA-2 (NCT01740427) and PALOMA-3 (NCT01942135) clinical studies, the protocol and informed consent form were reviewed and approved by an Institutional Review Board or Independent Ethics Committee (e.g., WCG IRB and Schulman Associates IRB, Inc). Patients provided written informed consent to participate in these studies.

### Gene expression profiling

The EdgeSeq Oncology Biomarker (BM) Panel (HTG Molecular Diagnostics, Tucson, AZ, USA) was used for mRNA profiling, assessing 2534 cancer and related genes. Measurement of gene expression was performed blinded to the clinical information. RNA expression levels of gene targets in FFPE tissues were quantitated using targeted capture sequencing. Laboratory process and manufacturer protocols were followed to prepare samples. The NextSeq^®^ 500 System Whole-Genome Sequencing Solution sequencer (Illumina, San Diego, CA, USA) performed the sequencing.

### Molecular subtype classification

Given only HR + patients were included in the PALOMA-2 and PALOMA-3 cohorts, and the lack of large, diverse reference tumor sets profiled with the EdgeSeq Oncology platform, common classification schemes like PAM50^44^ could not be used, as it determines subtypes relative to a baseline of heterogeneous tumors either from the same cohort or comparable reference database. The Absolute Intrinsic Molecular Subtyping (AIMS) single sample predictor algorithm was applied to assign subtypes through a set of binary rules that compare expression measurements for pairs of genes from a single patient only^45^. The EdgeSeq Oncology BM Panel included genes for 42 of the 100 AIMS binary rules. We evaluated classification performance by downsampling The Cancer Genome Atlas (TCGA) breast cancer data set from genome-wide to those on the EdgeSeq Oncology Panel and found that the agreement with PAM50 classification was 77% using all genes versus 76% using only the EdgeSeq Oncology BM Panel genes, as previously reported^18^.

### Statistical analysis

After gene expression data were quantile normalized and log2 transformed, Cox regression analysis was performed to investigate (1) potential interaction (dependency) between biomarker levels in terms of gene expression and treatment effect in terms of PFS (data cutoff date: May 31, 2017 for PALOMA-2 and October 23, 2015 for PALOMA-3): coxph(Surv(PFS) ~ gene expression * treatment arm) and (2) potential association between biomarker levels and PFS within a treatment arm: *coxph(Surv(PFS) ~ gene expression)* (Fig. 1). Gene expression was used as a continuous variable unless otherwise noted. Data normalization and Cox analysis were performed independently for PALOMA-2 and PALOMA-3 cohorts. Inter-trial concordance of interaction/association coefficient values was assessed by (1) Pearson correlation coefficient and its *P* value (null hypothesis of no relationship) and (2) the chance probability of obtaining at least the observed number of top biomarker candidates (nominal *P* ≤ 0.05) in common as determined by cumulative hypergeometric probability distribution. Kaplan-Meier survival curves were used for visualization, with gene expression dichotomized by median or quartile (quartile 1 vs quartile 4).

Unsupervised hierarchical clustering was performed with gene expression/PFS association measures (Cox coefficients) across all four treatment arms (palbociclib plus letrozole and placebo plus letrozole from PALOMA-2; palbociclib plus fulvestrant and placebo and fulvestrant from PALOMA-3). Distance metric was defined by Pearson correlation ($1 - \frac{{cov\left( {X,Y} \right)}}{{\sigma _X\sigma _Y}}$ where cov is the covariance and σ is the standard deviation), while average linkage was used for clustering. Principal component analysis (PCA) was performed using Partek^®^ Genomics Suite^®^ 7.0 (St Louis, MO, USA).

For gene set/signature analysis, enrichment analysis of PALOMA-2/3–specific and common genes associated with PFS from the palbociclib combination arm was performed using cumulative hypergeometric statistics^46^ and gene sets from Molecular Signatures Database^47^, with Benjamini-Hochberg false discovery rate (FDR)^48^ to account for multiplicity. Sample-level signature score was computed using the gene set variation analysis algorithm^49^ followed by statistical analysis as described above for gene biomarkers.

### Transcriptome analysis of breast cancer cell lines

MCF7 and T47D breast cancer cells (ATCC, Manassas, VA, USA) were cultured in RPMI 1640 medium (Gibco-Life Tech, Grand Island, NY, USA) supplemented with 10% fetal bovine serum (Sigma, St. Louis, MO, USA) and penicillin-streptomycin (Gibco-Life Tech, Grand Island, NY, USA) at 37 °C and 5% CO_2_. Cells were passaged routinely at a ratio of 1:3 or 1:4 every 3–4 days using trypsin/EDTA. After treatment with either palbociclib (200 nM) or the vehicle as control for one or seven days, they were harvested and profiled in biological duplicates. Whole transcriptome RNA sequencing was performed by Biomiga (San Diego, CA, USA). The 50-bp paired end reads were mapped by the bowtie2 algorithm^50^ and quantified using RSEM package^51^. Gene set enrichment analysis (GSEA) was performed with Signal2Noise metric and default parameters^47^.

### Reporting summary

Further information on research design is available in the Nature Research Reporting Summary linked to this article.

## Supplementary information

Supplementary figure 1a, fig 1b, fig 2, fig 3

REPORTING SUMMARY

## Data Availability

Gene expression data have been deposited into GEO (GSE133394 and GSE128500 for PALOMA-2 and −3 respectively). Upon request, and subject to certain criteria, conditions and exceptions (see https://www.pfizer.com/science/clinical-trials/trial-data-and-results for more information), Pfizer will provide access to individual de-identified participant data from Pfizer-sponsored global interventional clinical studies conducted for medicines, vaccines and medical devices (1) for indications that have been approved in the US and/or EU or (2) in programs that have been terminated (i.e., development for all indications has been discontinued). Pfizer will also consider requests for the protocol, data dictionary, and statistical analysis plan. Data may be requested from Pfizer trials 24 months after study completion. The de-identified participant data will be made available to researchers whose proposals meet the research criteria and other conditions, and for which an exception does not apply, via a secure portal. To gain access, data requestors must enter into a data access agreement with Pfizer.
