# Supplementary material for: Comparative biomarker analysis of PALOMA-2/3 trials for palbociclib
Source: NPJ Precis Oncol. 2022 Aug 16;6:56. doi: 10.1038/s41698-022-00297-1 (PMC9381541; doi:10.1038/s41698-022-00297-1)
Supplement: Supplementary file 1 — Supplementary figure 1a, fig 1b, fig 2, fig 3 [file 41698_2022_297_MOESM1_ESM.pdf]

# Comparative biomarker analysis of PALOMA-2/3 trials for palbociclib

Zhou Zhu, Nicholas C. Turner, Sherene Loi, Fabrice André, Miguel Martin, Véronique Diéras, Karen A. Gelmon, Nadia Harbeck, Cathy Zhang, Joan Q. Cao, Zhengming Yan, Dongrui R. Lu, Ping Wei, Todd L. VanArsdale, Paul A. Rejto, Xin Huang, Hope S. Rugo, Sibylle Loibl, Massimo Cristofanilli, Richard S. Finn, Yuan Liu[1]

Supplementary Material

**Supplementary Table 1.** Top candidates with nominal interaction P-value ≤0.05 in both PALOMA-2 and -3 trials.

| Gene | PALOMA-2 | PALOMA-3 |
|---|---|---|
| SP1 | Relative sensitivity | Relative sensitivity |
| TMEM45B | Relative sensitivity | Relative sensitivity |
| AK1 | Relative resistance | Relative resistance |
| GNAZ | Relative resistance | Relative resistance |
| MFNG | Relative resistance | Relative resistance |
| PINX1 | Relative sensitivity | Relative resistance |
| SRC | Relative sensitivity | Relative resistance |
| ABCC12 | Relative resistance | Relative sensitivity |
| CRP | Relative resistance | Relative sensitivity |

**Supplementary Table 2.** Cox proportional hazards regression analysis statistic for association of *ESR1* expression and PFS. Positive coefficient indicates that higher expression is associated with shorter PFS; negative coefficient indicates that higher expression is associated with longer PFS. Expression value was used as a continuous variable in the analysis. ET=endocrine therapy; PAL=palbociclib; PFS=progression-free survival.

| **Cox Regression Coefficient (*P* Value)** | **ET** | **PAL + ET** |
|---|---|---|
| PALOMA-2 | –0.111 (*P*=0.0457) | –0.172 (*P*=0.00192) |
| PALOMA-3 | –0.0734 (*P*=0.240) | –0.149 (*P*=0.00783) |
| PALOMA-3 (postmenopausal only) | –0.0441 (*P*=0.514) | –0.213 (*P*=0.000343) |

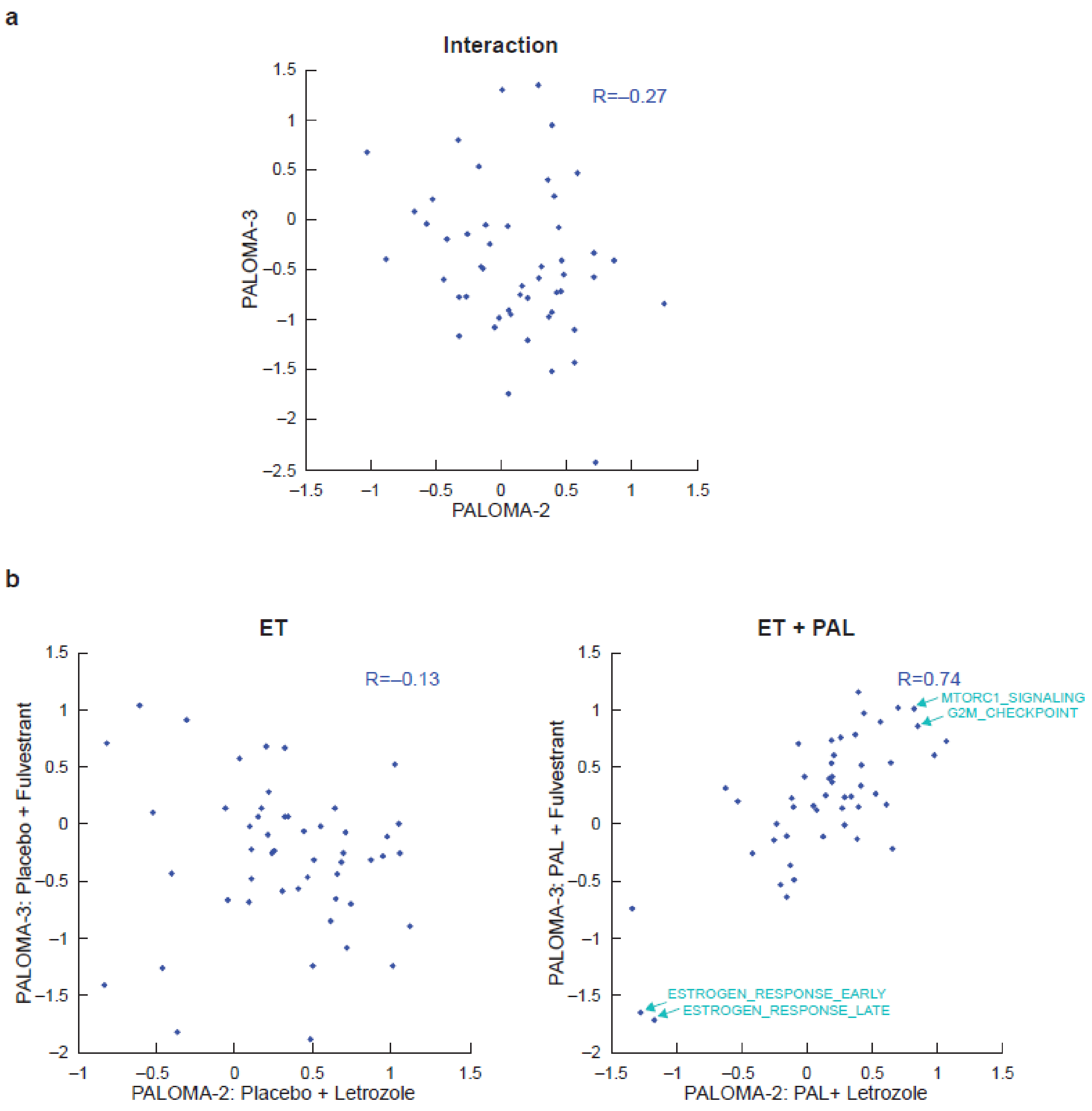


**Supplementary Figure 1:** Lack of correlation in predictive biomarker pattern between PALOMA-2 and PALOMA-3 appears driven by the poor concordance in the ET arm. Each data point corresponds to a MSigDB [47] hallmark gene set representing well-defined biologic states or processes. R value in the plots refers to Pearson correlation coefficient. **(a)** Comparison of signature expression/treatment effect interaction (dependency) in predicting PFS across the 2 trials. **(b)** Comparison of signature expression/PFS association within each treatment arm (ET, left panel; ET + palbociclib,

right panel) across the 2 trials. ET=endocrine therapy; PAL=palbociclib; PFS=progression-free survival.

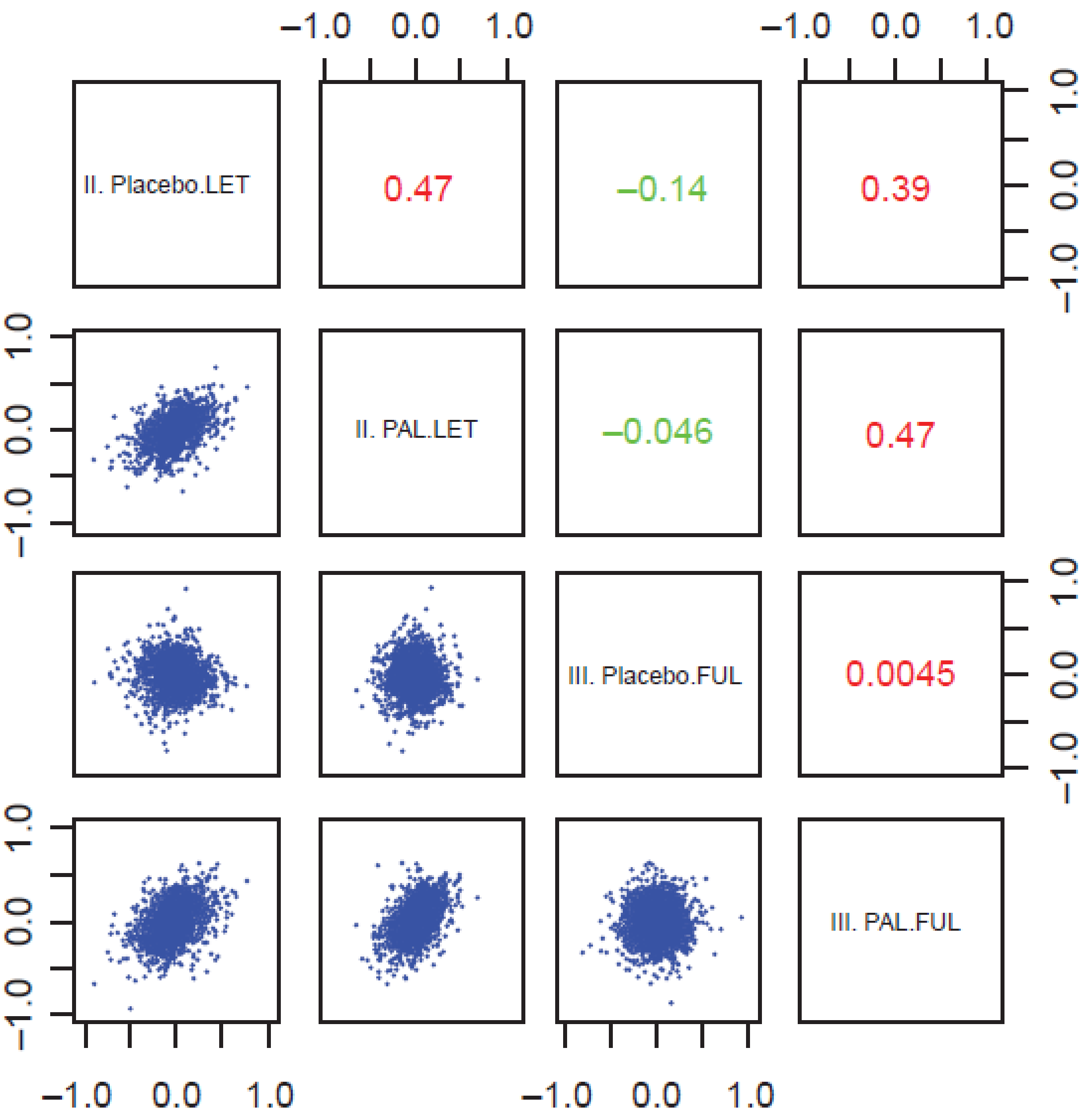


**Supplementary Figure 2:** Pairwise correlations of gene expression/PFS association pattern among the four treatment arms from PALOMA-2 (II) and PALOMA-3 (III) show that the palbociclib combination arms from both trials share strong similarity with each other as well as with the ET arm of advanced treatment-naive population from PALOMA-2, and all three are poorly related to the ET arm of the endocrine-resistant population from PALOMA-3. The numeric values in the plot correspond to Pearson correlation coefficient, with color indicating directionality (red=positive correlation;

green=negative correlation). The respective scatterplots are also shown. ET=endocrine therapy; FUL=fulvestrant; LET=letrozole; PAL=palbociclib; PFS=progression-free survival.

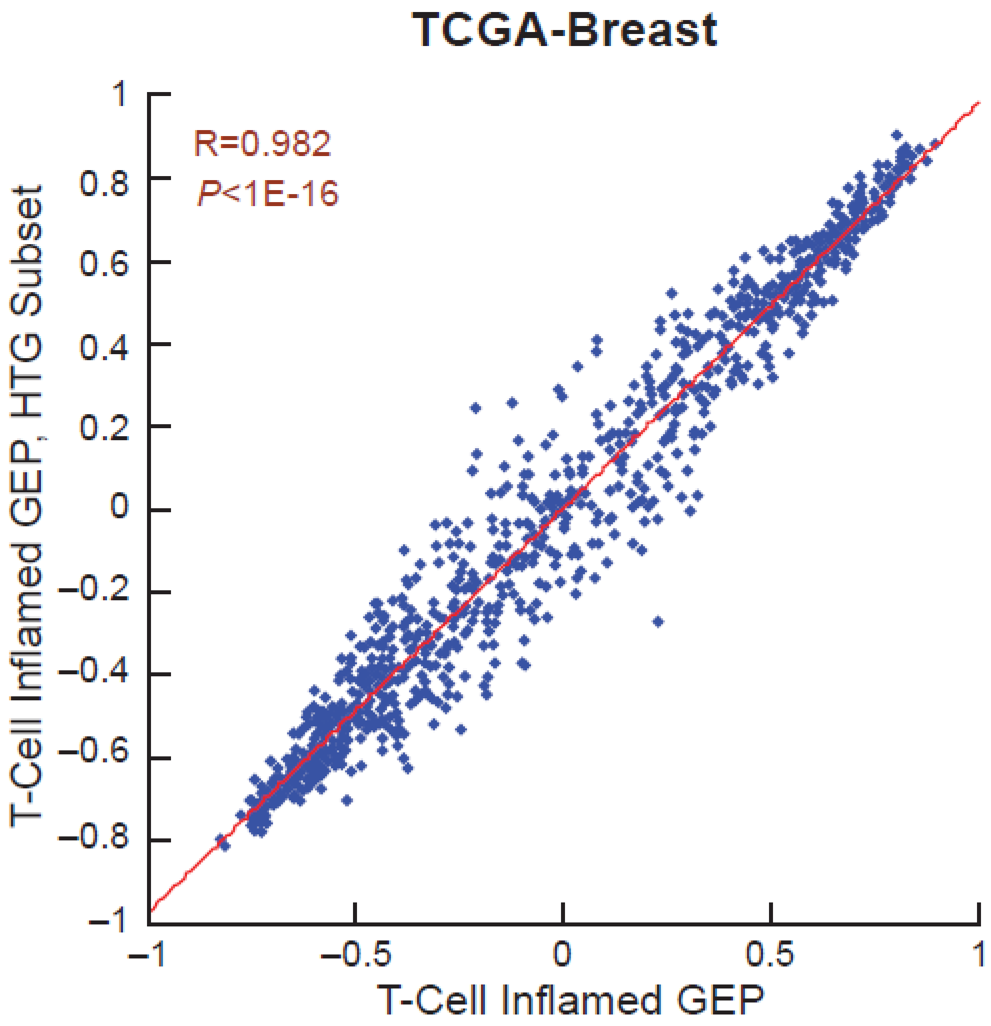


**Supplementary Figure 3:** Downsampling of an 18-gene signature of T-cell–inflamed TME [23] to the 13-gene subset profiled by EdgeSeq Oncology HTG Panel using TCGA breast cohort showed little impact of the 5 missing genes on its quantification. Signature score for each sample was computed using gene set variation analysis (GSVA) [49] with the full set and EdgeSeq Oncology HTG subset. R value refers to Pearson correlation coefficient. GEP=gene expression profile; TCGA=The Cancer Genome Atlas; TME=tumor environment.
